# Attenuating hypoxia driven malignant behavior in glioblastoma with a novel hypoxia-inducible factor 2 alpha inhibitor

**DOI:** 10.1038/s41598-020-72290-2

**Published:** 2020-09-16

**Authors:** Jaclyn J. Renfrow, Michael H. Soike, James L. West, Shakti H. Ramkissoon, Linda Metheny-Barlow, Ryan T. Mott, Carol A. Kittel, Ralph B. D’Agostino, Stephen B. Tatter, Adrian W. Laxton, Mark B. Frenkel, Gregory A. Hawkins, Denise Herpai, Stephanie Sanders, Jann N. Sarkaria, Glenn J. Lesser, Waldemar Debinski, Roy E. Strowd

**Affiliations:** 1 Department of Neurological Surgery, Wake Forest Baptist Medical Center, Winston-Salem, NC USA; 2 Department of Radiation Oncology, Wake Forest Baptist Medical Center, Winston-Salem, NC USA; 3 Department of Pathology, Wake Forest Baptist Medical Center, Winston-Salem, NC USA; 4 Foundation Medicine, Inc., Morrisville, NC USA; 5 Brain Tumor Center of Excellence, Wake Forest Comprehensive Cancer Center, Winston-Salem, NC USA; 6 Department of Biostatistical Sciences, Wake Forest Baptist Medical Center, Winston-Salem, NC USA; 7 Department of Biochemistry, Wake Forest Baptist Medical Center, Winston-Salem, NC USA; 8 Department of Cancer Biology, Wake Forest Baptist Medical Center, Winston-Salem, NC USA; 9 Department of Radiation Oncology, Mayo Clinic, Rochester, MN USA; 10 Department of Internal Medicine – Section on Hematology and Oncology, Wake Forest Baptist Medical Center, Winston-Salem, NC USA; 11 Department of Neurology, Wake Forest Baptist Medical Center, Winston-Salem, NC USA; 12 One Medical Center Drive, Department of Neurosurgery, Wake Forest Baptist Medical Center, Winston-Salem, NC 27157 USA

**Keywords:** CNS cancer, Cancer therapy

## Abstract

Hypoxia inducible factor (HIFs) signaling contributes to malignant cell behavior in glioblastoma (GBM). We investigated a novel HIF2α inhibitor, PT2385, both in vitro*,* with low-passage patient-derived cell lines, and in vivo, using orthotopic models of glioblastoma. We focused on analysis of HIF2α expression in situ, cell survival/proliferation, and survival in brain tumor-bearing mice treated with PT2385 alone and in combination with standard of care chemoradiotherapy. HIF2α expression increased with glioma grade, with over half of GBM specimens HIF2α positive. Staining clustered in perivascular and perinecrotic tumor regions. Cellular phenotype including proliferation, viability, migration/invasion, and also gene expression were not altered after PT2385 treatment. In the animal model, PT2385 single-agent treatment did improve median overall survival compared to placebo (*p* = 0.04, n = 21) without a bioluminescence correlate (t = 0.67, *p* = 0.52). No difference in animal survival was seen in combination treatment with radiation (RT)/temozolomide (TMZ)/PT2385 (*p* = 0.44, n = 10) or mean tumor bioluminescence (t 1.13, *p* = 0.32). We conclude that HIF2α is a reasonable novel therapeutic target as expressed in the majority of glioblastomas in our cohort. PT2385 as a single-agent was efficacious in vivo, however, an increase in animal survival was not seen with PT2385 in combination with RT/TMZ. Further study for targeting HIF2α as a therapeutic approach in GBM is warranted.

## Introduction

Oxygen regulation is a critical cellular process that has been highly conserved throughout evolution. Hypoxia inducible factors (HIFs) are transcription factors that mediate the cellular response to hypoxia. The first HIF described in the literature was HIF1α which was identified by its role as a regulator of the gene erythropoietin. Subsequent discovery has given rise to a family of transcription factors involved in the cellular response to hypoxia including HIF2α and HIF3α^1–3^. These transcription factors are critical to the cellular response to hypoxia throughout the body and contribute to the mechanisms underlying stroke, heart attacks, inflammatory diseases, and cancer^4,5^.

In cancer, hypoxia is linked to malignant tumor behavior by driving cell proliferation, promoting angiogenesis, enhancing migration, and facilitating chemoradiation resistance^6–10^. Despite a well-described link between hypoxia and malignancy, targeting HIFs in oncology has not led to therapeutic advances in the clinic. Downstream processes involving *HIF* sensitive genes related to malignant behavior include angiogenesis via VEGF and EPO, cell division via CCND1, and cellular metabolism via GLUT. HIF1α is ubiquitously expressed in both tumor and normal tissue. This limits the therapeutic specificity of targeting HIF1α and risks unintended side effects as a result of a drug’s effects on HIF1α expression in non-tumor tissues^11,12^. HIF2α appears to be a more attractive target for the following reasons: its expression is more specific to tumor tissues, it mediates states of chronic hypoxia found in tumors, and specific inhibitors to target HIF2α exist^13^. There is a limited understanding of the function of HIF3α and at this time no specific oncologic therapeutic potential has been demonstrated yet. Thus, HIF2α is the candidate HIF isoform for further investigation into its role in malignancy and as a therapeutic target.

Glioblastoma is a malignancy defined by regions of pseudopalisading necrosis, which represents a hypoxic core enveloped by tumor^14^ making it attractive for investigational testing of anti-hypoxia therapy. In gliomas, HIF2α is expressed in glioma cells but not in normal neural progenitors or glia^15,16^ and may be clinically relevant as HIF2α expression in the REMBRANDT glioma database (n = 834) showed higher HIF2α expression in tumors correlates with increased grade of malignancy and poorer patient survival^15^. HIF2α is tumor specific and correlates with patient outcome in gliomas.

Despite the hypothetical merits of HIF2α as a therapeutic target in gliomas, translational investigations have been limited by the lack of clinically applicable inhibitors. Magnetic resonance spectroscopy and crystallography studies in the early 2000s defined the HIF2α/ARNT-binding pocket^17,18^. This geometric information informed a small-molecule screen which identified 130 potential compounds and ultimately resulted in developing PT2385, a first-in-class, orally available HIF2α transcriptional inhibitor^13^. PT2385 was originally developed for targeting renal cell carcinoma with Von Hippel Lindau (*VHL)* mutations and recently began preliminary phase I human studies^19,20^. This agent also has promising blood brain barrier permeability with a brain:plasma ratio of 0.9 in rats (Personal Communication, Peloton Therapeutics), thus allowing for preclinical evaluation of HIF2α inhibition in glioblastoma.

In the present study, we sought to (1) evaluate in situ protein expression of HIF2α in gliomas, (2) link increased HIF2α expression to hypoxia in glioblastoma cells in vitro and test the effect of a clinically available HIF2α inhibitor, PT2385, on cell survival and proliferation, and (3) explore the activity of PT2385 as a single agent and then in combination with standard of care chemoradiotherapy for GBM.

## Methods

### Immunohistochemistry

Fifty-seven gliomas (grade II-IV) (samples from Wake Forest Brain Tumor Center of Excellence and Mayo Clinic) were stained for HIF2α by immunohistochemistry with approval from the Wake Forest Baptist Medical Center Institutional Review Board. Informed consent from subjects was obtained per protocol and all experiments were performed in accordance with relevant guidelines and regulations. Epitopes were retrieved with Tris EDTA pH 9.0, detected with a HIF2α antibody (sc-13596, Santa Cruz, CA) followed by a secondary antibody conjugated with horseradish peroxidase (Vector Labs, Burlingame, CA). HIF2α staining was visualized by Nova Red (Vector Labs), counterstained with hematoxylin, dehydrated, cleared and mounted with permount. Semiquantitative visual scoring of HIF2α stained and complementary hematoxylin and eosin stained slides were performed by a board-certified neuropathologist to estimate the location of staining (i.e. perivascular, perinecrotic, or other), cell source (i.e. tumor cells, endothelial, astrocytes, etc.), and percent of tumor cells staining positive. High HIF2α expression was considered to be tumor samples with greater than 10% of cells staining for HIF2α and low HIF2α expression was considered to be tumor samples with less than 1% of cells staining for HIF2α. The grouping of expression was then converted into a numeric score so that > 10% positive cells were rated 3, between 1 and 10% positive cells rated 2, and < 1% positive cells rated 1, and no positive cells rated 0.

### Immunoblotting

After 72 h of culturing cells in hypoxic conditions (1% O2) (Xvivo 3 by Biospherix, Parish, NY) and normoxic conditions (ThermoForma Series II CO2 Incubator by Fisher Scientific, Hampton, NH) cell lysates were prepared in RIPA buffer with protease and phosphatase inhibitors (Sigma) and separated by 10% SDS-PAGE. Western blotting was performed as previously described^21^. Primary antibodies included HIF2α antibody (sc-46691, Santa Cruz, CA) and β-Actin (Sigma, St. Louis, MO).

### Cell viability

For cell viability assays, PT2385-treated (10 μM in DMSO) and sham-treated cells in normoxic and hypoxic conditions were analyzed using the LIVE/DEAD Viability/Cytotoxicity Kit, for mammalian cells (ThermoFisher, Carlsbad, CA). Briefly, 2 × 10^5^ cells were plated in 24-well plates in triplicate and drug treatments started 12–24 h after plating and continued for a period of three days. RPMI-1640 mammalian cell culture media supplemented with 10% fetal bovine serum was removed and 200 mL of the calcein AM/ethidium homodimer-1 solution was added to the well and allowed to incubate at room temperature for 30 min. The plates were analyzed using a BMG AbTech Fluostar Optima luminescence plate reader and corresponding photographs taken with Olympus IX70 inverted fluorescence microscope.

### Cell proliferation

For cell proliferation assays, 1 × 10^6^ cells from *10* μM PT2385-treated and sham-treated cells in normoxic and hypoxic conditions were fixed in 70% ethanol and washed with PBS. The cell pellet was resuspended in 0.5 mL of FxCycle PI/ RNAse solution (ThermoFisher, Carlsbad, CA) and incubated in the dark at room temperature for 30 min. Cells were analyzed for cell cycle fractions on a BD Accuri C6 Analyzer Flow Cytometer using 488-nm excitation and 585-nm collection filters. Data was analyzed using FCS express6 software from DeNovo (Glendale, CA).

### Nu/Nu mouse intracranial injections

Patient-derived GBM cell lines (MGMT/IDH status unknown) were established in culture as previously described^22^. Primary cells in culture were then transfected with a lentiviral vector containing luciferase/RFP under the same promoter (GenTarget Inc, San Diego, CA) and selected for infected cells using blasticidin. Cells were assayed for luciferase activity and red fluorescent protein expression.

Approximately 6 × 10^5^ cells were implanted into the right frontal lobes of 6 and 8-week-old athymic *Nu/Nu* female mice obtained from Charles River Laboratories. All applicable international, national, and institutional guidelines for the care and use of animals were followed. All procedures performed in studies involving animals were in accordance with the ethical standards of the institution at which the studies were conducted and approved by the Wake Forest Baptist Medical Center Institutional Animal Care and Use Committee (IACUC). BTCOE 4795 was used as this cell line consistently formed tumors which on histology demonstrated necrosis and stained positive for HIF2α. Mice were anesthetized with 0.1 mL/20 g mouse weight IP ketamine/xylazine (87.5 mg/kg ketamine and 12.5 mg/kg xylazine) and placed on a small-animal head-holding frame. A midline scalp incision exposed a burr-hole site 2-mm to the right and 1-mm anterior to lambda. A 27-gauge needle attached to a sterile Hamilton syringe (Hamilton, Reno, NV) was stereotactically inserted 3-mm below the cortical surface and cells were injected into the deep white matter in a volume of 7μL over 5 min. The syringe was removed over an additional minute after completion of injection. The scalp was closed with an interrupted 6-0 prolene suture. Mice recovered on a heating pad and returned to their cages after waking from anesthesia. Treatments are detailed below. Brains of euthanized mice were collected, fixed in 10% buffered formalin and either paraffin embedded or preserved in tissue-freezing medium (Triangle Biomedical Sciences, Durham, NC).

### Single-arm PT2385 treatment

A stock solution of PT2385 was prepared in 30% PEG400, 10% ethanol, 0.5% tween 80, and 0.5% methylcellulose. PT2385 was administered 10 mg/kg PO BID in half the animals; the other half receiving vehicle treatment. As per company recommendations on dosing scheduled the drug was administered in 21 day on drug followed by 7 days off drug cycles repeated until endpoint met.

### Radiation and temozolomide treatment

Animals were randomized using a random number generator (GraphPad, La Jolla, CA) into treatment groups consisting of RT/TMZ/PT2385 and RT/TMZ/Placebo. Tumors developed for two weeks after implantation. Baseline bioluminescence imaging was obtained to confirm presence of tumor. Animals without tumor or tumor signal indicative of metastases or spinal cord involvement were excluded. TMZ was administered at 50 mg/kg (capsules dissolved in sterile water) orally on days 1–5 of a 28-day cycle. PT2385 was administered as described above. Radiation was delivered at a dose of 20 Gy in four fractions prescribed to 3-mm depth using the OrthoVolt platform (Precision X-ray, North Branford, CT). Mice were anesthetized and placed in the left lateral recumbent position with a 7 mm aperture to encompass the right frontal lobe, avoiding the oral cavity and oropharynx. On days of radiation, TMZ and PT2385 or placebo were administered one hour prior to radiation delivery.

### Bioluminescent imaging

Tumor growth was monitored by evaluating bioluminescence using the IVIS Lumina II imaging system (Xenogen Corporation, Alameda, CA). Animals received an intraperitoneal (i.p.) injection of d-luciferin (150 mg/kg, stock solution 15 mg/mL in sterile PBS, Goldbio, St. Louis, MO). After 15 min, animals were anesthetized with 3% isofluorane until non-responsive, and then placed into the imaging chamber. Bioluminescent imaging acquisition was collected at 120 s. Data was analyzed based on total photon flux emission (photons) in the region of interest over the intracranial space using Living Image software (Xenogen Corp., Alameda, CA). Individual mouse data was also normalized such that the treatment values were divided by the highest pre-treatment luminescent value^23,24^.

### Real-time PCR

Plated cells were treated with vehicle and PT2385 (Peloton Therapeutics Inc.) in both a conventional and hypoxia (1% O2) cell culture incubators for twenty-four hours. Cells were pelleted, and RNA extracted using Ambion RNA extraction Kit (Life Technologies, Carlsbad, CA). cDNA was then synthesized using a high-capacity RNA-to-cDNA kit (Applied Biosystems, Carlsbad, CA). cDNA along with master mix (ThermoFisher Scientific, was loaded into a commercially available 96-well plate containing validated hypoxia pathway TaqMan assays which included dried PCR primers and TaqMan**®** probes (ThermoFisher Scientific, Grand Island, NY) and qPCR was performed using an ABI 7500 machine. More information on the commercially available hypoxia pathway qPCR plate can be found at https://www.thermofisher.com/order/catalog/product/4414090. Results were analyzed for gene expression fold change.

### RNA purification and RNAseq

BTCOE 4795 tumor punches from extracted mouse brains (pathologist verified) were homogenized using the Bead Ruptor 24 (Omni International, Tulsa, OK). Approximately 10–50 mg of tissue was placed into a 1.4 mm ceramic bead tube with 1 ml QIAzol lysis reagent. The tissue sample tube was processed on the Bead Ruptor for 1 cycle at a speed of 4.7 m/s for 20 s. The homogenized lysates were extracted for total RNA using the RNeasy Microarray Tissue Mini kit (Qiagen, Venlo, Netherlands). Extracted RNA was DNase-treated and purified using the RNA Clean and Concentrator-5 kit (Zymo Research, Irvine, CA) and assessed for RNA quality using an Agilent 2100 Bioanalyzer and the RNA 6000 Nano Kit (Agilent Technologies, Santa Clara, CA).

Total RNA from 10 samples was used to prepare cDNA libraries using the Illumina® TruSeq Stranded Total RNA with Ribo-Zero Gold Preparation kit (Illumina Inc., San Diego, CA). RIN values for the RNA samples ranged from 9.6 to 10. Briefly, 750 ng of total RNA was rRNA depleted followed by enzymatic fragmentation, reverse-transcription and double-stranded cDNA purification using AMPure XP magnetic beads. The cDNA was end repaired, 3′ adenylated, with Illumina sequencing adaptors ligated onto the fragment ends, and the stranded libraries were pre-amplified with PCR. The library size distribution was validated, and quality inspected using a Fragment Analyzer (Advanced Analytical, Santa Clara, CA). The quantity of each cDNA library was measured using the Qubit 3.0 (Thermo Fisher, Waltham, MA). The libraries were pooled and sequenced to a target read depth of 40 M reads per library using single-end (SE) 1 × 75 bp sequencing on the Illumina NextSeq 500.

### RNAseq data analysis

The alignment and quality control of RNA-Seq data followed the pipeline developed by The NCI's Genomic Data Commons(GDC,https://gdc.cancer.gov/). The quality assessment was performed using FASTQC (https://www.bioinformatics.babraham.ac.uk/projects/fastqc/) on the pre-alignment and RNA-SeQC^25^ and Picard tools (https://broadinstitute.github.io/picard/) on the post-alignment. The sequence alignment was performed using a two-pass method using STAR2^26^. Read Counts analysis were performed using SummarizedExperiment of DESeq2^27^ and the count data normalized using BetweenLaneNormalization function of EDASeq^28^ using upper-quartile (UQ) normalization. The factors of unwanted variation were estimated using empirical control genes, e.g., least significantly differentially expressed (DE) genes based on a first-pass DE analysis performed prior to RUVg normalization^29^. Lastly, differential expression analysis was performed as implemented by DESeq2 by adding the factors of unwanted variation into design of DESeq2 in order to remove unwanted variation. The data from this analysis is publically available on GEO starting on September 10, 2020 using the link https://urldefense.proofpoint.com/v2/url?u=https-3A__www.ncbi.nlm.nih.gov_geo_info_linking.html&d=DwIEAg&c=yzGiX0CSJAqkDTmENO9LmP6KfPQitNABR9M66gsTb5w&r=amoisFLV_GDPJ2a20llMYDoNyc5dLQ81QU5P990ku58&m=TQfyxDWeYthcaGu5ekPM0wPMDIREs7eLBYGxNPTfQRY&s=0sjRPwkxUxPQAAsoY0sSLpPo4Gb__Haey4VC9fH-1qU&e=.

### IPA analysis

The *p* value < 0.01 and log2 ratio change <  −1 and > 1 to get DEGs for IPA. The reference set for IPA is Ingenuity Knowledge Base, where both direct and indirect relationships were used for both networks and upstream regulator analysis. Of the top canonical pathways found by this analysis, the first, second, and fourth were explored in greater depth due to their z-scores. The top three networks of interactions between members of the dataset were also expanded and explored here. The functional pathway analyses were generated through the use of IPA (QIAGEN Inc., https://www.qiagenbioinformatics.com/products/ingenuity-pathway-analysis)^30^.

### Gene set enrichment analysis (GSEA)

GSEA pathway enrichment analysis was performed using the database for annotation, visualization and integrated discovery (DAVID) v6.8 (https://david.ncifcrf.gov/). A false discovery rate (FDR) threshold of *p* ≤ 0.05 was used to determine significance level.

### Survival analysis

Kaplan–Meier estimates of overall survival (OS) and median survival were calculated for the mice treated with vehicle versus single agent PT2385 and for mice randomized to RT/TMZ/Placebo versus RT/TMZ/PT2385.

For immunohistochemistry clinical data from TMA analysis, HIF2α expression from previously untreated glioblastoma was stratified by high expression (score of 2–3) vs low expression (0–1) to determine the hazard for death for HIF2α overexpression. Kaplan–Meier estimates were performed to determine survival. Group comparisons of OS rates were performed using the log-rank test with *p* values < 0.05 considered to be statistically significant. SAS version 9.4 (SAS Institute, Cary, NC) was used to conduct all survival analyses.

## Results

### HIF2α analysis in human glioma specimens

**Figure 1:**
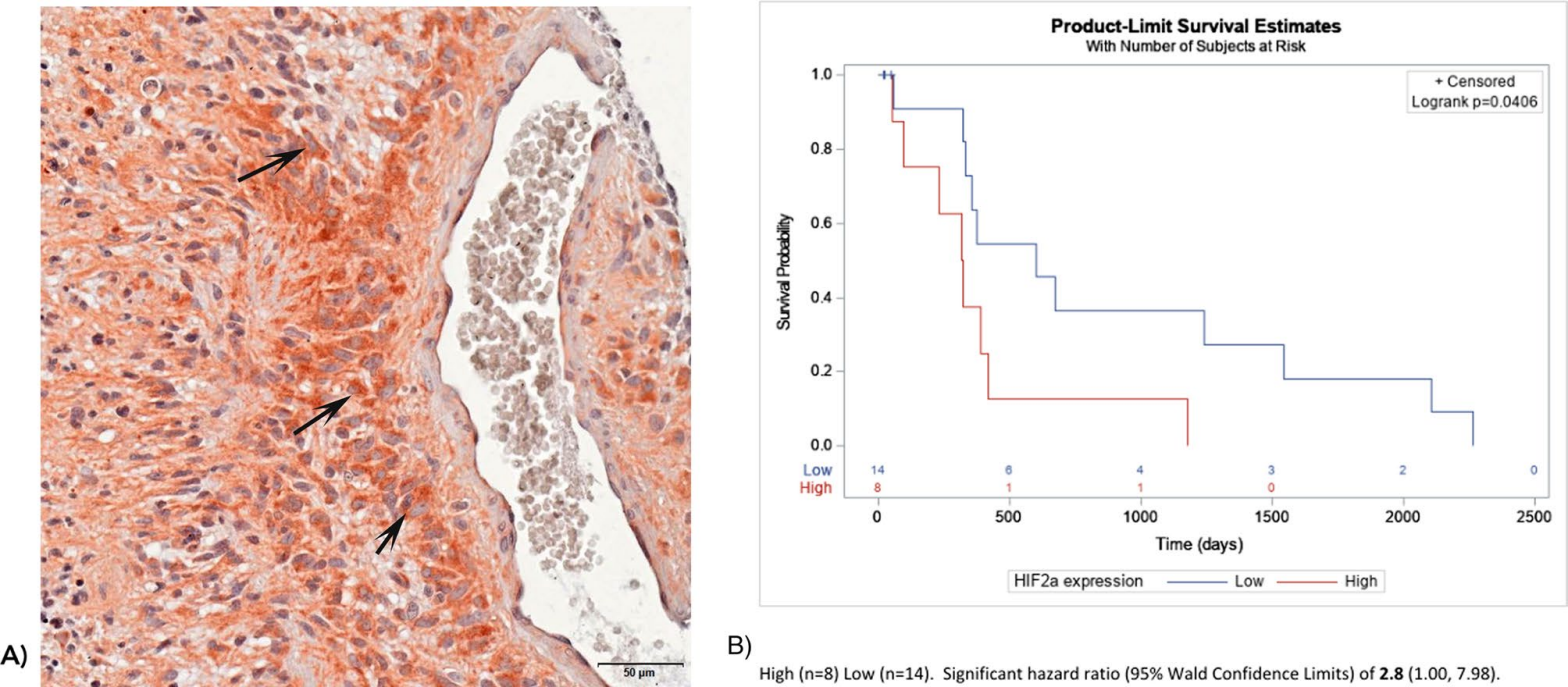
(**A**) Immunohistochemistry on a paraffin embedded glioblastoma sample stained for HIF2α expression demonstrating perivascular staining with arrows indicating representative cells expressing HIF2α. (**B**) Kaplan Meier curve demonstrating higher abundance of HIF2α was associated with an increased hazard for mortality (HR: 2.8, 95% CI 1.00–7.98, log-rank *p* = 0.04).

**Figure 2:**
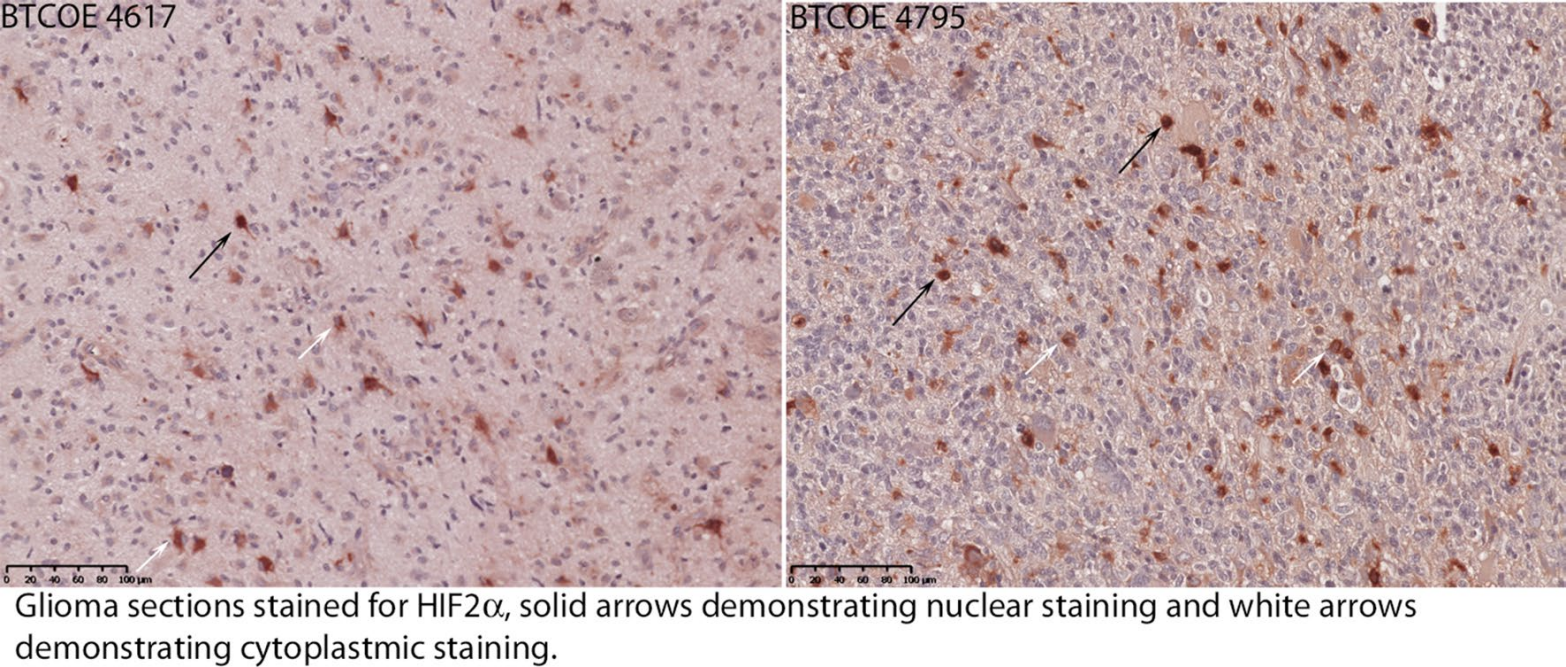
Immunohistochemistry on paraffin embedded glioblastoma samples stained for HIF2α expression demonstrating the observation of both nuclear (black arrows) and cytoplasmic (white arrows) staining with the majority of staining observed to be cytoplasmic.

In situ HIF2α protein expression was studied in four WHO grade II, eleven WHO grade III astrocytomas, and 42 WHO grade IV glioblastomas. HIF2α was present in 0/4 grade II tumors, 72% (8/11) of the grade III gliomas, and 64% (27/42) of the GBMs (Grade IV) with higher percentages of HIF2α staining cells noted in recurrent tumor samples compared to newly diagnosed GBMs (Supplementary Fig. 1). Staining was specific to tumor cells and rarely observed in peritumoral monocytic cells. HIF2α was primarily expressed in perivascular and perinecrotic regions (Fig. 1A). In untreated GBMs (N = 22), a higher abundance of HIF2α was associated with an increased hazard for mortality (HR: 2.8, 95% CI 1.00–7.98, log-rank *p* = 0.04, Fig. 1B). Within individual cells staining was mainly cytoplasmic with rare observations of nuclear staining (Fig. 2).

### HIF2α in glioma cell culture

To document a direct link between HIF2α expression and hypoxia, two low-passage, adult patient-derived GBM cell lines (BTCOE 4536 and BTCOE 4795, Supplementary Table 1) were cultured in both normoxic and hypoxic conditions for 72 h and compared to a renal cell carcinoma cell line (Caki1) known to upregulate HIF2α in hypoxia. Both BTCOE 4536 and BTCOE 4795 demonstrated prominent upregulation of HIF2α protein expression when cultured in hypoxic conditions compared to normoxia (Supplementary Fig. 2).

**Figure 3:**
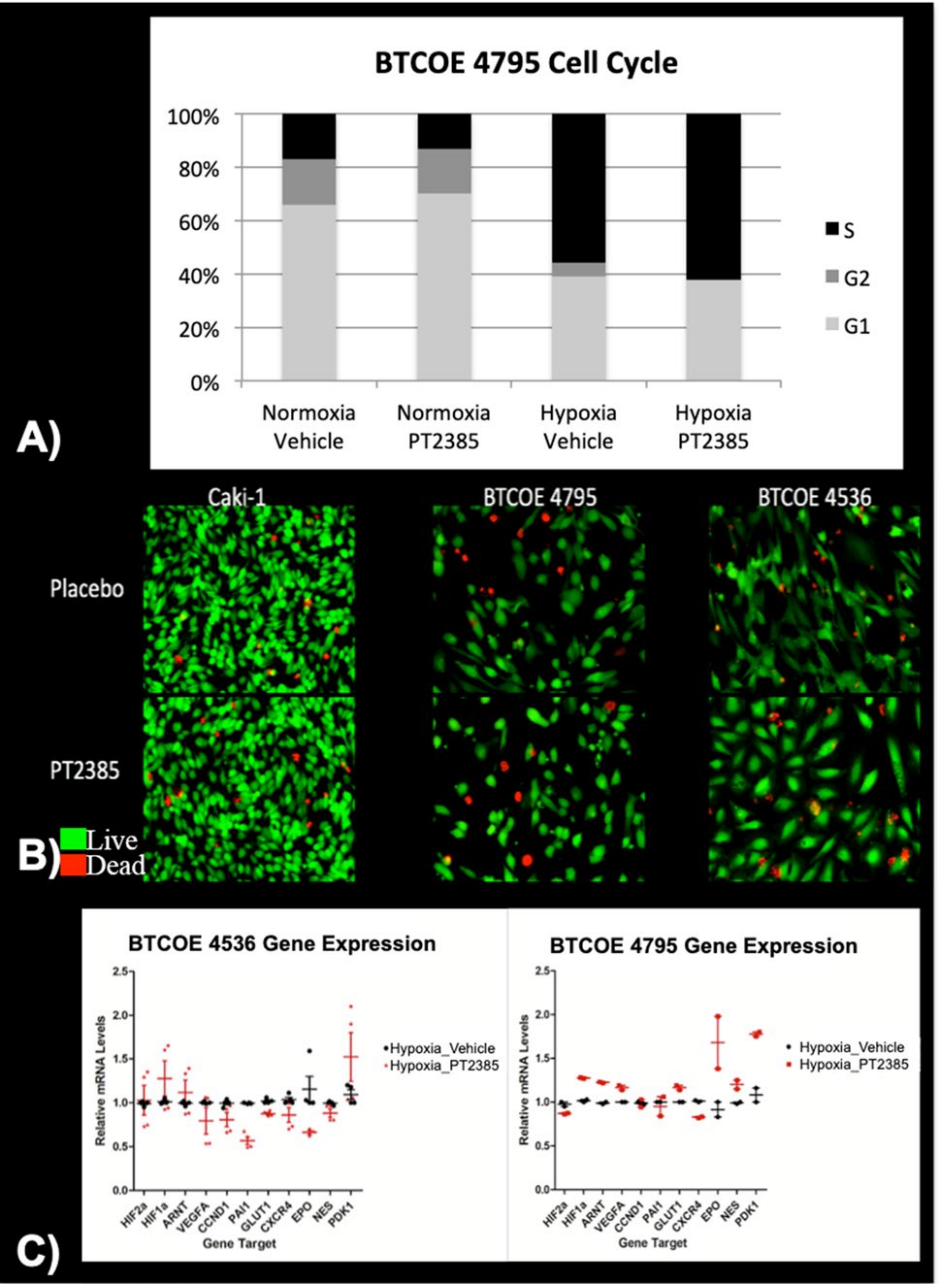
(**A**) Cell cycle analysis in glioma cells treated with either 10 μM PT2385 or vehicle in normoxic and hypoxic conditions for 72 h demonstrate no measurable difference between treatment groups. (**B**) Fluorescent microscopy of cell viability testing in renal cell carcinoma and glioma cells treated with either PT2385 or vehicle in hypoxic conditions with viable cells appearing green and dead cells appearing red demonstrate no visual difference between treatment groups, (**C**) Real-time PCR gene expression fold changes for hypoxia pathway HIF2α downstream genes were decreased after PT2385 treatment in the BTCOE 4536 cell line and unchanged in the BTCOE 4795 cell line.

To study the effects of HIF2α inhibition in GBM, we utilized an oral HIF2α inhibitor, PT2385, developed for human use. In vitro testing of PT2385 on glioma cells did not demonstrate a measurable effect on cell proliferation and cell viability measured through cell cycle analysis (Fig. 3A, B). Real-time PCR measurements of gene expression for hypoxia pathway genes also demonstrated variable changes in downstream HIF2α targets with PT2385 treatment (Fig. 3C). In BTCOE 4795 cells, downstream HIF2α gene expression does not appear to be downregulated with PT2385 treatment. Instead, HIF1a appears to be upregulated, which may be driving the persistent expression of downstream genes. The BTCOE 4536 GBM cell line demonstrated downregulation of VEGF, CCND1, PAI, GLUT, CXCR4, and EPO indicating reduced expression of downstream targets of HIF2α.

### HIF2α in mouse glioma model

**Figure 4:**
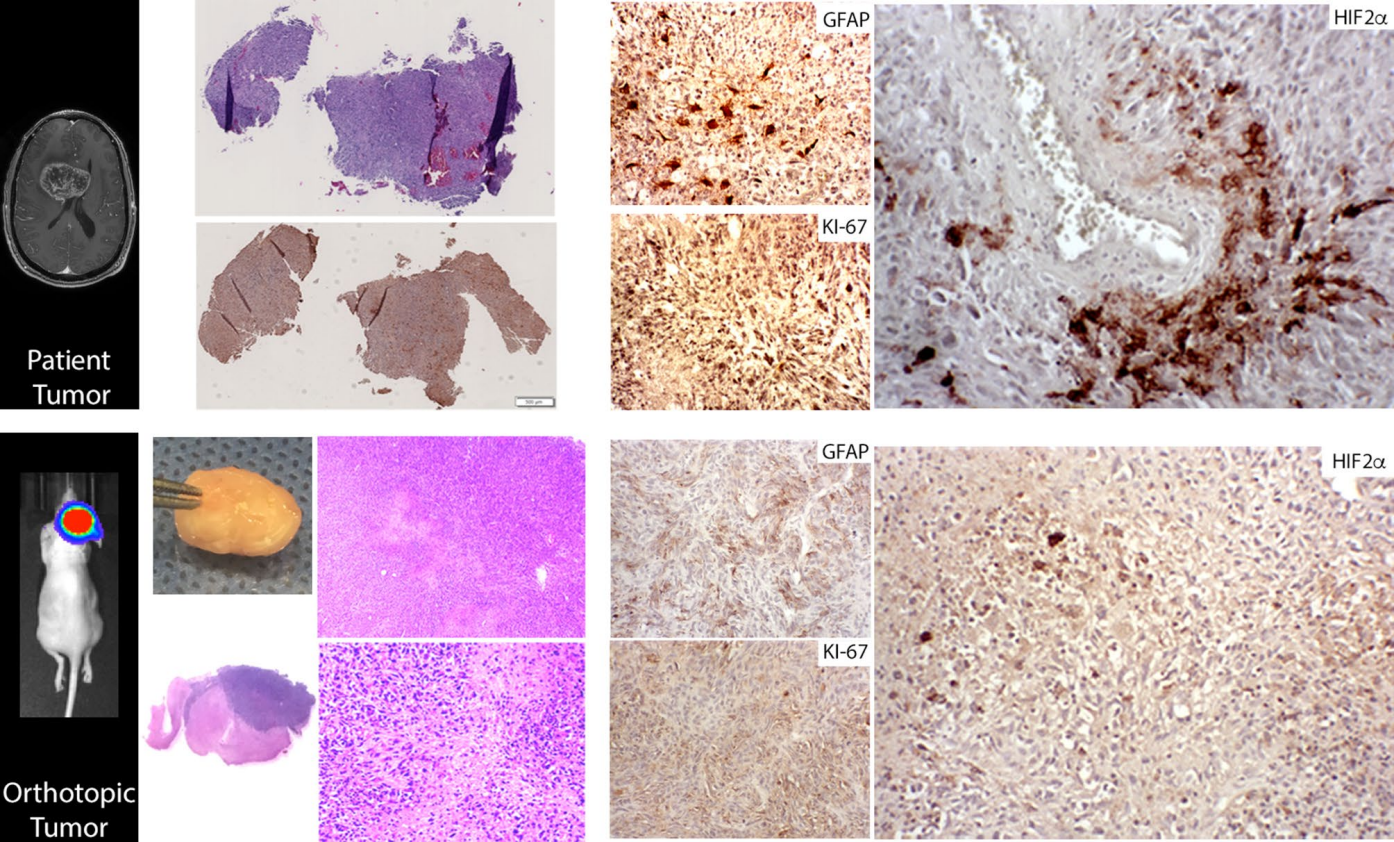
Original H&E histology of a glioblastoma sample stained for HIF2α using immunohistochemistry along with GFAP and Ki-67 (upper panels). Once this tumor was implanted into mice formation of tumors were verified with bioluminescence and grossly with corresponding histology using H&E, which demonstrated the model retained necrotic features and glioma markers including HIF2α, GFAP, and Ki-67 (lower panels).

**Figure 5:**
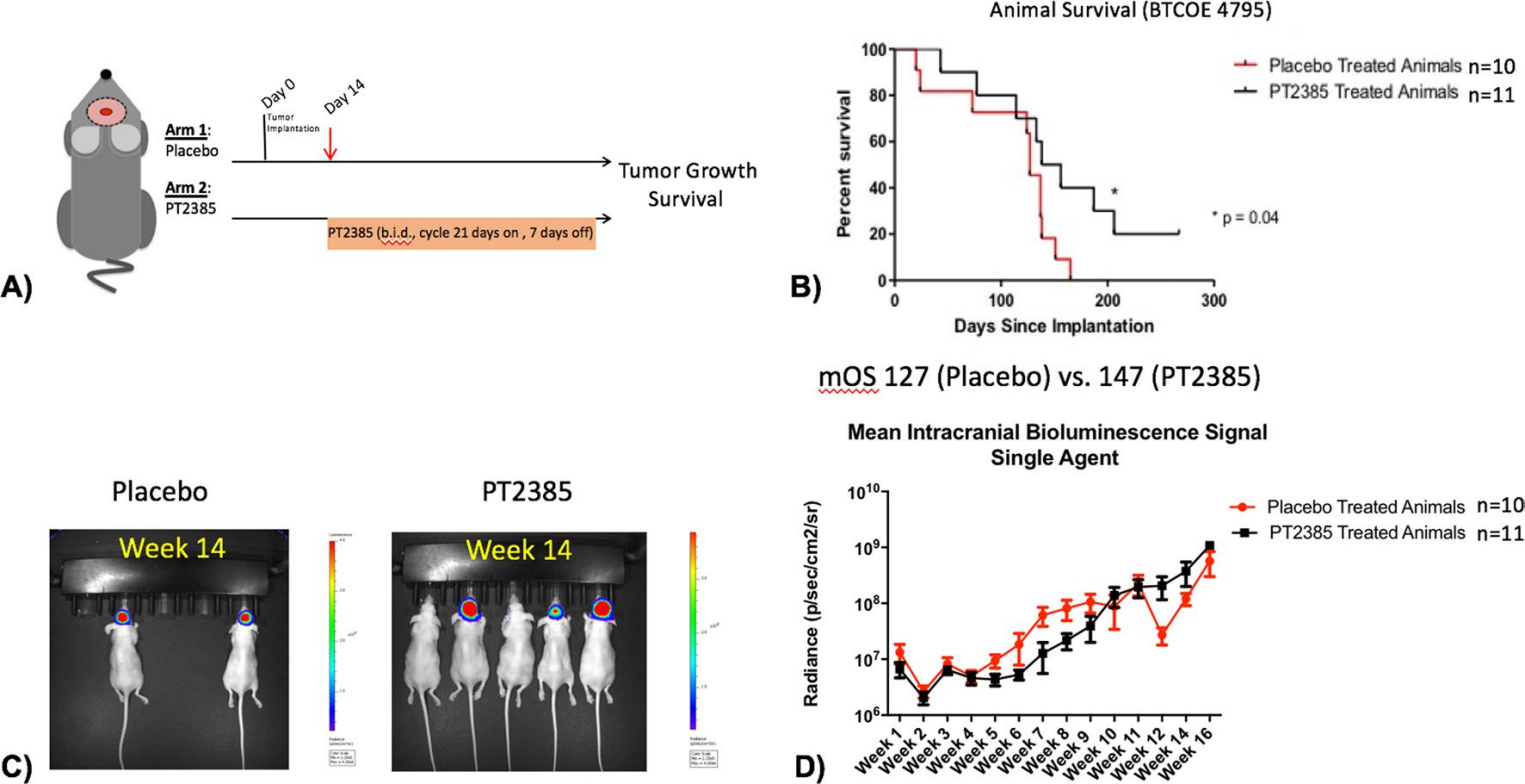
(**A**) Experimental design for single agent (10 mg/kg PO BID PT2385 in 21 days on/7 days off cycles) studies (using model BTCOE 4795). (**B**) Kaplan Meier survival curve demonstrating animals receiving PT2385 survived longer than animals receiving placebo (*p* = 0.04). (**C**) Representative bioluminescent images of a cage receiving placebo treatment and a cage receiving PT2385 treatment after 14 weeks with more animals alive and smaller tumor burdens amongst PT2385 treated animals. (**D**) Graph of bioluminescent values during 16 weeks of consecutive imaging show similar signal intensities when treatment with vehicle is compared to PT2385 treated animals (t = 0.67, *p* = 0.52).

To investigate the consequences of HIF2α inhibition in vivo, first a single-agent study utilizing an orthotopic patient-derived GBM model using the BTCOE 4795 cell line was performed (Fig. 4). Following two bioluminescent imaging studies confirming intracranial tumorigenicity of this cell line by fourteen days, twenty-one mice were randomized to single-agent PT2385 or vehicle (Fig. 5A). Median overall survival for mice treated with PT2385 was significantly longer than untreated (147 vs. 127 days, *p* = 0.0477, Fig. 5B). Representative images of mice from both groups at 14 weeks of treatment depict more mice living in the PT2385 group with less tumor burden on average (Fig. 5C). Bioluminescence imaging demonstrated no statistically significant differences between the groups (t = 0.67, *p* = 0.5181) (Fig. 5D/Supplementary Fig. 5).

**Figure 6:**
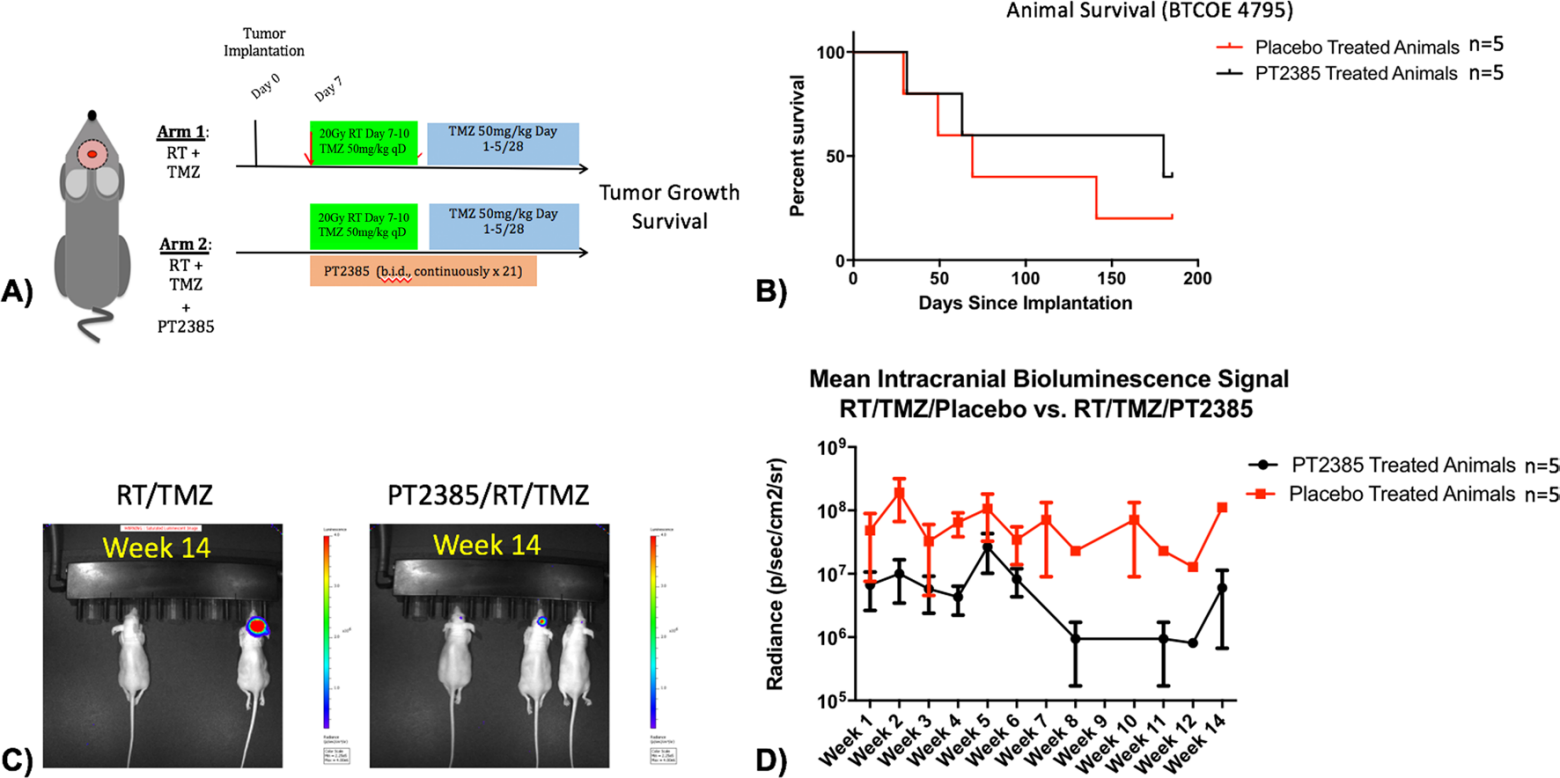
(**A**) Experimental design for chemoradiation (50 mg/kg PO qD TMZ during radiation followed by 5 days on/23 days off cycles) combined with PT2385 (10 mg/kg PO BID PT2385 in 21 days on/7 days off cycles) studies (using model BTCOE 4795). (**B**) Kaplan Meier survival curve demonstrating no significant difference in survival in animals treated with RT/TMZ/PT2385 versus RT/TMZ/Placebo (*p* = 0.44). (**C**) Representative bioluminescent images of a cage receiving placebo treatment and a cage receiving PT2385 treatment after 14 weeks with more animals alive and smaller tumor burdens amongst PT2385 treated animals. (**D**) Graph of bioluminescent values during 14 weeks of consecutive imaging demonstrate no significant difference between RT/TMZ/Placebo animals compared to RT/TMZ/PT2385 (t 1.13, *p* = 0.32).

Next, PT2385 was studied in combination with standard of care therapies for GBM: radiation and temozolomide using the BTCOE 4795 cell line (Fig. 6A). In a cohort of 10 animals, the median survival of RT/TMZ/Placebo treated animals was 69 days versus 180 days in animals treated with RT/TMZ/PT2385 (*p* = 0.44, Fig. 6B). A second expansion cohort of 13 animals were implanted to follow and had a median survival of 83 days in the RT/TMZ/Placebo treated animals versus 69 days in animals treated with RT/TMZ/PT2385 (*p* = 0.54). Representative images after 14 weeks of treatment demonstrate at this timepoint more mice living with smaller tumor burdens in animals treated with RT/TMZ/PT2385 relative to RT/TMZ/Placebo (Fig. 6C), yet a definitive signal in terms of extended survival or lower bioluminescence was not observed in the RT/TMZ/PT2385 treatment cohort (t 1.13, *p* = 0.32) (Fig. 6D/Supplementary Fig. 5).

To investigate genome wide expression changes comparing PT2385 treated tumors to placebo treated tumors from the single-agent study (BTCOE 4795), animals sacrificed from the single-agent studies with their tumors explanted were analyzed by RNAseq analysis at the time of animal death. Ingenuity Pathway Analysis (IPA) revealed upregulation of four pathways in the PT2385 treated animals compared to placebo including: opioid signaling, CREB signaling, synaptic long-term depression, and glutamate receptor signaling (Supplementary Fig. 4). The opioid pathway showed the greatest change with a 5 log-fold upregulation in PT2385 treated tumors compared to placebo. Notable pathway genes upregulated included protein kinase C (*PKC*) and calmodulin dependent protein kinases (*CaMK*). Phosphatidylinositol-4,5-bisphosphate 3-kinase catalytic subunit gamma (*PI3KG*) expression was down-regulated. A secondary analysis was performed using the GSEA methodology via DAVID. The top three enriched pathways with the greatest fold changes included voltage-gated potassium channel activity, potassium channel, and anterior/posterior pattern specification (Supplementary Table 2).

## Discussion

Hypoxia is a pervasive feature of the glioma microenvironment and is linked to both tumor aggression as well as therapeutic resistance^16,31–35^. Targeting HIF2α represents a novel therapeutic approach in cancer, including GBM. In this study, we show that HIF2α is expressed in the majority of GBMs, possesses a strong biologic rationale as a therapeutic target, and targeting HIF2α extended animal survival in single-agent in vivo studies.

HIF2α protein expression geographically clustered in perivascular and perinecrotic niches that are thought to give rise to treatment resistance (Fig. 1A). Staining was mainly cytoplasmic but there were a few observations of nuclear staining. Prior fluorescent microscopy co-localization studies suggest that these regions are niches for glioma stem-like cells and type 2 tumor associated macrophages that may facilitate therapeutic resistance^15,36^. In prior studies, HIF2α expression, and not HIF1a, was linked to patient survival in the REMBRANDT database^15^. This was confirmed on a protein expression level in our cohort of untreated GBM patients. HIF2α expression was also investigated as part of the Phase II Bevacizumab/Irinotecan recurrent GBM trial and patients with high carbonic anhydrase 9 (CA9) and HIF2α expression demonstrated the worst survival outcomes whereas, conversely, patients with low CA9 and HIF2α expression had the best clinical outcomes^37^. In our specimens, HIF2α expression correlated with increasing grade of malignancy and was more prominent in samples of recurrent disease. Complementing this finding, a prior flow cytometry study of temozolomide-resistant gliomas noted HIF2α upregulation was universal and increased with time^34^. These studies support the notion that HIF2α clinically correlates with tumor grade and patient survival.

Prior in vitro work using short-hairpin RNA knockdown of HIF2α in glioma stem-like cells demonstrated an anti-tumor response through reduced tumorsphere formation, reduced expression of GSC oncogenes, and extended survival in brain tumor bearing mice^15,38^. A lack of pharmacologic agents limited further clinical investigation until the recent advent of a specific HIF2α inhibitor, PT2385. The data presented here expands upon prior studies by establishing HIF2α as a potential therapeutic target in GBM using a clinically available agent.

Patient-derived glioma cell lines cultured in hypoxic conditions demonstrated an increase in HIF2α expression (Fig. 2). When GBM cell lines were treated with PT2385 no differences in cell cycle (Fig. 3) nor migration/invasion (data not shown) were noted compared to placebo treatment (Fig. 3). These results are consistent with viability and proliferation experiments with PT2385 in renal cell carcinoma cell lines^20^ as well as prior work demonstrating HIF2α knockdown does not affect in vitro cell proliferation^39,40^. It is possible that cell culture drug metabolism along with the culture medium including serum may contribute to these results. However, gene expression in BTCOE 4536 demonstrated downregulation of HIF2α downstream targets (and not HIF1α downstream targets, PDK1) following PT2385 treatment. On the other hand, we did not see these changes in BTCOE 4795 cells. This is thought to result, in part, from a compensatory increase in HIF1α expression which was observed in this cell line (Fig. 3C). HIF1α then may have been driving the preserved expression of the downstream gene targets despite HIF2α downregulation. Such a scenario may predict a resistance mechanism to HIF2α inhibition treatment strategies. Overall, HIF2α inhibition via PT2385 in GBM was similar to prior findings in renal cell carcinoma which indicated that cell proliferation and viability are unaffected in vitro. Data from renal cell flank tumor growth in animals treated with PT2385 suggest a more cytostatic than cytotoxic effect, lending PT2385 to further combinatorial regimens^20^.

In vivo testing demonstrated single agent efficacy of PT2385 with 20 days of extended median survival compared to placebo treated animals in an orthotopic intracranial model (Fig. 4). Bioluminescent tumor imaging demonstrated lower signal in mice treated with PT2385 compared to placebo mice followed for a period of 10 weeks. RNAseq analysis of single-agent treated tumors was intended to identify genetic changes induced by PT2385 treatment to elucidate the mechanism of efficacy, however, the genetic changes likely represent a resistance signature. Given all tumors progressed at the time of animal death, these results may, inform mechanisms and strategies to circumvent treatment resistance. The opioid signaling pathway was upregulated fivefold following PT2385 treatment and may represent an alternative mechanism to activate malignant growth factor pathways^41^. Single-agent studies demonstrated PT2385 extends survival over placebo in mice bearing intracranial tumors.

The incorporation of PT2385 into standard of care treatment with RT/TMZ regimen in the mice did not demonstrate an additive effect on survival. Tumor burden estimated by bioluminescence also demonstrated no significant difference between the groups. It is worthy to note that PT2385 is cytostatic in mechanism, not cytotoxic, meaning that reduced tumor bioluminescence may not be anticipated with such a mechanism. Animal dropout may also have contributed to the inability to resolve a statistically significant difference on imaging that did exist. Additional possible additional factors affecting overall survival in this group include known toxicities of RT/TMZ such as thrombocytopenia and oropharyngeal irritation/failure though theoretically this should have been present evenly amongst the treatment groups. It is also possible the addition of PT2385 contributed to additive toxicities with TMZ, including anemia (a known human toxicity), meaning that dosing may not be optimal. Finally, the gene status of MGMT, a known predictor of TMZ response^42^, was not known in this mouse model.

There are several limitations to this study. While prior studies demonstrate HIF2α exerts an anti-tumor effect in vitro on tumorsphere formation and gene expression, HIF2α inhibition by a clinically available agent, PT2385, did not reproduce these findings on patient derived low-passage adherent glioma cell lines. This suggests that for its activity the drug may require interaction with tumor microenvironment. Alternatively, additional factors including the time and severity of in vitro hypoxia and limited functional assays for analysis may have also contributed to a lack of findings. PT2385 did appear to have modest efficacy as a single agent in intracranial tumors. The incorporation of PT2385 into standard of care treatment with RT/TMZ regimen in the mice did not demonstrate an additive effect on survival. We also note conclusions cannot be drawn whether PT2385 has any synergy with either single agent TMZ or RT alone. Tumor burden estimated by bioluminescence also demonstrated no significant difference between the groups. Possible additional factors affecting overall survival in this group include known toxicities of RT/TMZ such as thrombocytopenia and oropharyngeal irritation/failure though theoretically this should have been present evenly amongst the treatment groups. It is also possible the addition of PT2385 contributed to additive toxicities with TMZ, including anemia (a known human toxicity), meaning that dosing may not be optimal. Finally, the gene status of MGMT, a known predictor of TMZ response, was not known in this mouse model. These observations also suggest future studies may include possible alternative strategies for HIF2α downregulation that may include other novel agents and even possibly a combination downregulation of partial HIF1α along with HIF2α to mitigate potential compensatory effects.

HIF2α represents an attractive therapeutic target in GBM with a strong biologic rationale warranting further study. Here, we characterize glioma expression of HIF2α and evaluate the therapeutic potential of targeting HIF2α using a clinically available agent, PT2385. Intracranial bioluminescent imaging in mice suggests a possible cytostatic effect and detectable single-agent efficacy in overall survival demonstrated targeting HIF2α may be part of a rational combinational therapy for further exploration. A Phase II clinical study (NCT03216499) investigating downregulating the hypoxia pathway through PT2385 is underway in patients with recurrent GBM.

## Supplementary information

Supplementary information
