# Supplementary material for: Attenuating hypoxia driven malignant behavior in glioblastoma with a novel hypoxia-inducible factor 2 alpha inhibitor
Source: Sci Rep. 2020 Sep 16;10:15195. doi: 10.1038/s41598-020-72290-2 (PMC7495485; doi:10.1038/s41598-020-72290-2)
Supplement: Supplementary file 1 — Supplementary information [file 41598_2020_72290_MOESM1_ESM.pdf]

Attenuating Hypoxia Driven Malignant Behavior in Glioblastoma with a Novel Hypoxia-Inducible Factor 2 Alpha Inhibitor

Jaclyn J. Renfrow[1,5], Michael H. Soike[2], James L. West[1], Shakti H. Ramkissoon[3,4], Linda Metheny-Barlow[2,5], Ryan T. Mott[3,5], Carol Kittel[6], Ralph B. D'Agostino[5,6], Stephen B. Tatter[1,5], Adrian W. Laxton[1,5], Mark B. Frenkel[1], Gregory A. Hawkins[7], Denise Herpai[5,8], Stephanie Sanders[5,8], Jann N. Sarkaria[9], Glenn J. Lesser[5,10], Waldemar Debinski[5,8], Roy E. Strowd[5,10, 11]

[1] Department of Neurological Surgery, Wake Forest Baptist Medical Center, Winston-Salem, NC
[2] Department of Radiation Oncology, Wake Forest Baptist Medical Center, Winston-Salem, NC
[3] Department of Pathology, Wake Forest Baptist Medical Center, Winston-Salem, NC
[4] Foundation Medicine, Inc., Morrisville, NC
[5] Brain Tumor Center of Excellence, Wake Forest Comprehensive Cancer Center, Winston-Salem, NC
[6] Department of Biostatistical Sciences, Wake Forest Baptist Medical Center, Winston-Salem, NC
[7] Department of Biochemistry, Wake Forest Baptist Medical Center, Winston-Salem, NC
[8] Department of Cancer Biology, Wake Forest Baptist Medical Center, Winston-Salem, NC
[9] Department of Radiation Oncology, Mayo Clinic, Rochester, MN
[10] Department of Internal Medicine – Section on Hematology and Oncology, Wake Forest Baptist Medical Center, Winston-Salem, NC
[11] Department of Neurology, Wake Forest Baptist Medical Center, Winston-Salem, NC

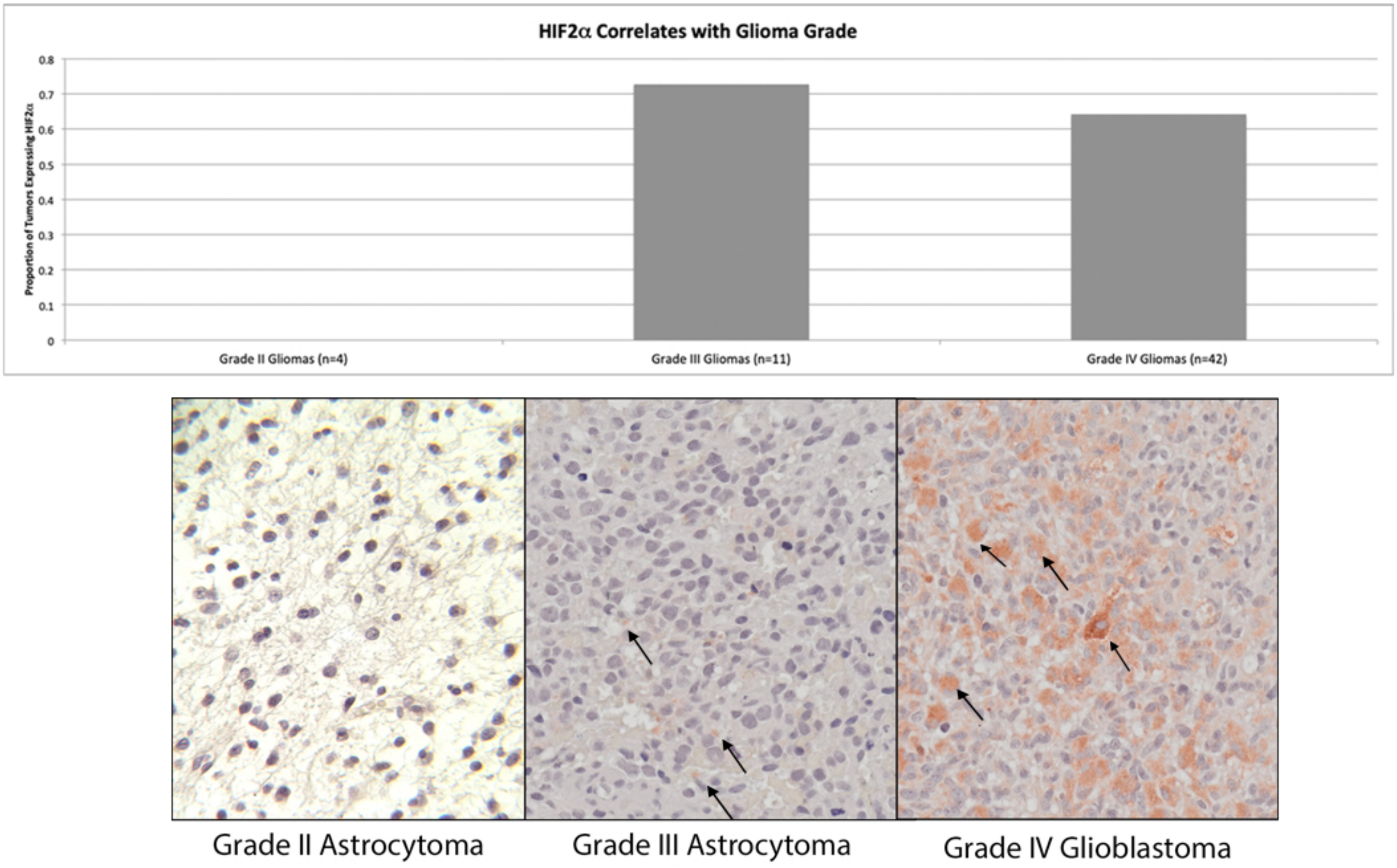
HIF2α Correlates with Glioma Grade
Proportion of Tumors Expressing HIF2α
0.8
0.7
0.6
0.5
0.4
0.3
0.2
0.1
0
Grade II Gliomas (n=4)
Grade III Gliomas (n=11)
Grade IV Gliomas (n=42)
Grade II Astrocytoma
Grade III Astrocytoma
Grade IV Glioblastoma

Supplementary Figure 1 Bar graph summarizing the presence of HIF2α in a cohort of increasing grades of gliomas (upper panel). Immunohistochemistry on human glioma samples of increasing grade for HIF2a demonstrating preferential staining in high grade gliomas with images at 40x magnification (lower panel).

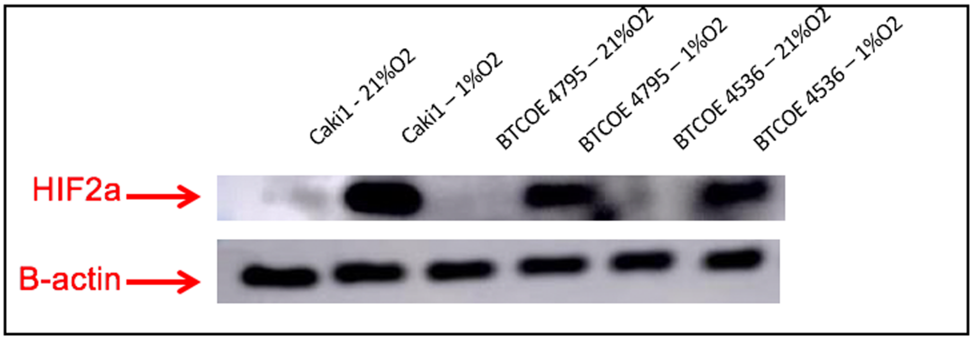
Caki1 - 21%O2
Caki1 – 1%O2
BTCOE 4795 – 21%O2
BTCOE 4795 – 1%O2
BTCOE 4536 – 21%O2
BTCOE 4536 – 1%O2
HIF2a
B-actin

Supplementary Figure 2 Western blot with protein isolates from Caki-1 (renal cell carcinoma cell line), BTCOE 4795 and BTCOE 4536 (patient-derived glioblastoma cell lines) stained for HIF2α expression in both conditions of normoxia (21% O2) and hypoxia (1% O2) for 72 hours with staining for β-Actin as a loading control. The gel for this blot was cut to facilitate primary antibody staining for HIF2α and β-Actin respectively and the bands from the corresponding molecular weights were cropped to create the figure. Hypoxia upregulates HIF2α protein expression in all three cell lines.

BTCOE 4795 Cell Cycle Analysis

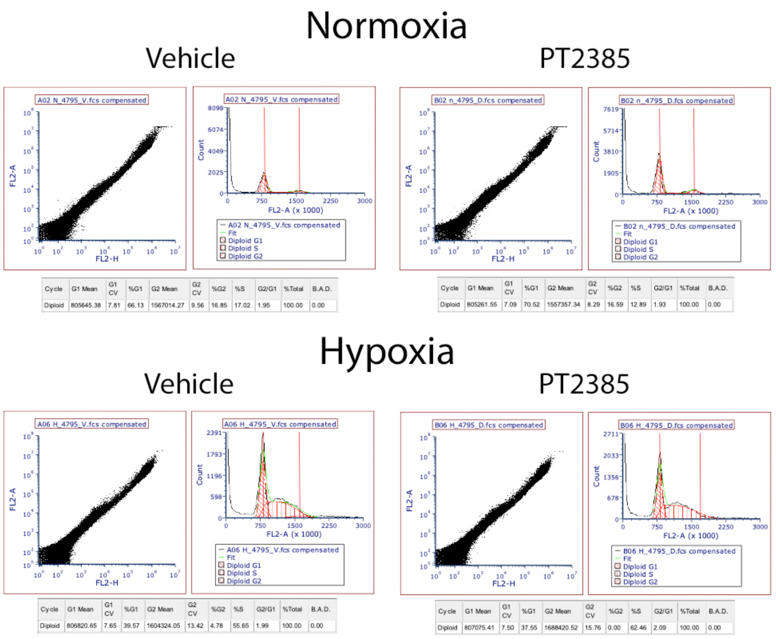

Normoxia – Vehicle

Cycle	G1 Mean	G1 CV	%G1	G2 Mean	G2 CV	%G2	%S	G2/G1	%Total	B.A.D.
Diploid	805645.38	7.81	66.13	1567014.27	9.56	16.85	17.02	1.95	100.00	0.00

Normoxia – PT2385

Cycle	G1 Mean	G1 CV	%G1	G2 Mean	G2 CV	%G2	%S	G2/G1	%Total	B.A.D.
Diploid	805261.55	7.09	70.52	1557357.34	8.29	16.59	12.89	1.93	100.00	0.00

Hypoxia – Vehicle

Cycle	G1 Mean	G1 CV	%G1	G2 Mean	G2 CV	%G2	%S	G2/G1	%Total	B.A.D.
Diploid	806820.65	7.65	39.57	1604324.05	13.42	4.78	55.65	1.99	100.00	0.00

Hypoxia – PT2385

Cycle	G1 Mean	G1 CV	%G1	G2 Mean	G2 CV	%G2	%S	G2/G1	%Total	B.A.D.
Diploid	807075.41	7.50	37.55	1688420.52	15.76	0.00	62.46	2.09	100.00	0.00

A)

BTCOE 4795 Cell Viability Analysis

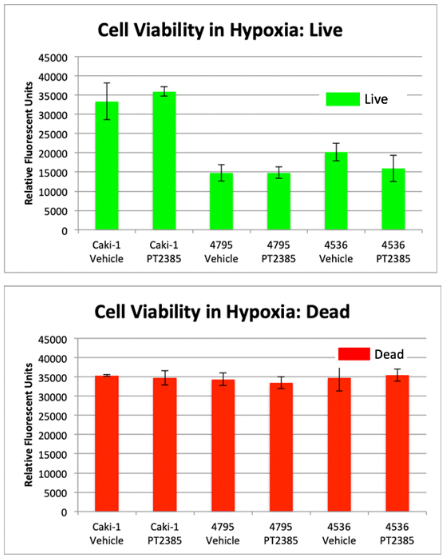

B)

Supplementary Figure 3 A) Cell cycle analysis scatter plot and histograms for summative graph depicted in Figure 3A. **B)** Numerically data for relative fluorescence measurements in the cell viability assay images represented in Figure 3B.

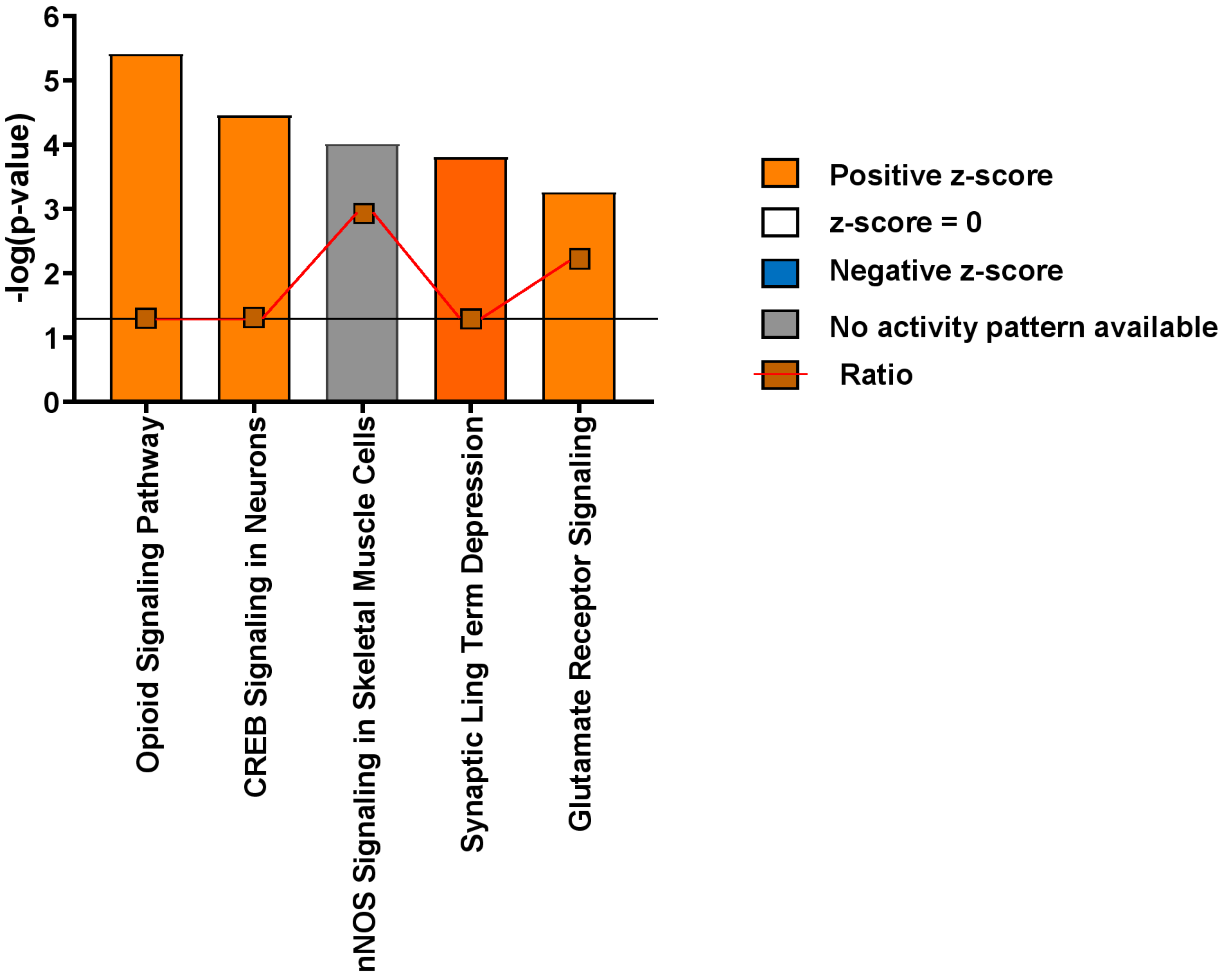
-log(p-value)
6
5
4
3
2
1
0
Opioid Signaling Pathway
CREB Signaling in Neurons
nNOS Signaling in Skeletal Muscle Cells
Synaptic Ling Term Depression
Glutamate Receptor Signaling
Positive z-score
z-score = 0
Negative z-score
No activity pattern available
Ratio

Supplementary Figure 4 Ingenuity Pathway Analysis (IPA) revealed upregulation of four pathways in the PT2385 (10 mg/kg PO BID PT2385 in 21 days on/7 days off cycles) treated animals compared to placebo including: opioid signaling, CREB signaling, synaptic long-term depression, and glutamate receptor signaling. The functional pathway analyses were generated through the use of IPA (QIAGEN Inc., https://www.qiagenbioinformatics.com/products/ingenuity-pathway-analysis)[30].

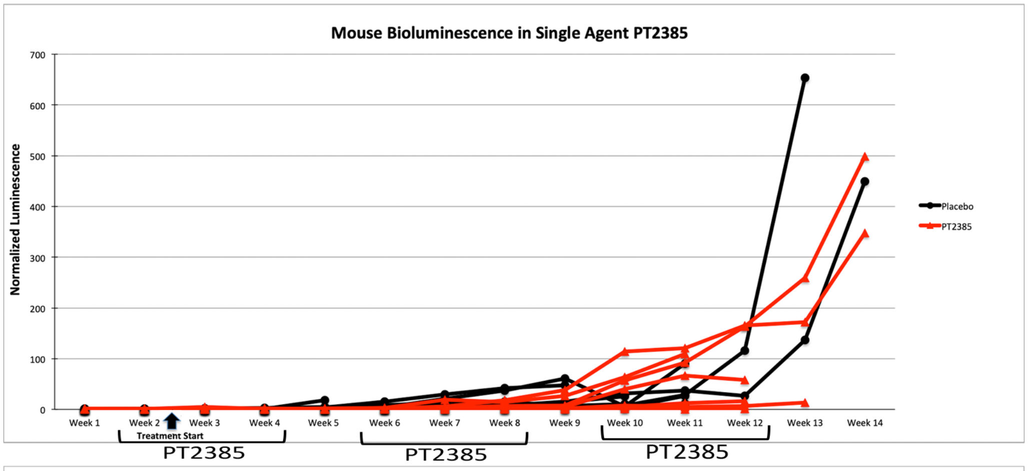
Mouse Bioluminescence in Single Agent PT2385
Normalized Luminescence
700
600
500
400
300
200
100
0
Week 1
Week 2
Week 3
Week 4
Week 5
Week 6
Week 7
Week 8
Week 9
Week 10
Week 11
Week 12
Week 13
Week 14
Treatment Start
PT2385
PT2385
PT2385
Placebo
PT2385

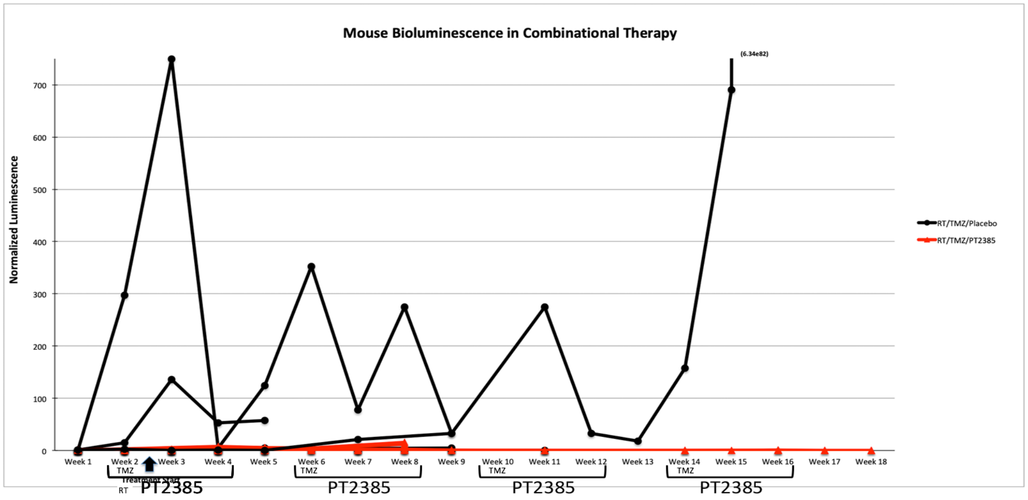
Mouse Bioluminescence in Combinational Therapy
Normalized Luminescence
(6.34e82)
700
600
500
400
300
200
100
0
Week 1
Week 2
Week 3
Week 4
Week 5
Week 6
Week 7
Week 8
Week 9
Week 10
Week 11
Week 12
Week 13
Week 14
Week 15
Week 16
Week 17
Week 18
TMZ
TMZ
TMZ
TMZ
RT
PT2385
PT2385
PT2385
PT2385
RT/TMZ/Placebo
RT/TMZ/PT2385

Supplementary Figure 5 Individual normalized bioluminescent values for mice undergoing the single agent study (upper panel) and the combinational study (RT/TMZ/PT2385) in the lower panel.

Supplementary Table 1

Cell Line	Age/Sex	Location	Specimen Source
BTCOE 4536	71 Female	Right Temporal	Glioblastoma – index resection
BTCOE 4795	43 Male	Right Frontal	Glioblastoma – first recurrence resection

Supplementary Table 2

Pathway	Representative Genes	Fold Enrichment	p-value	False Discovery Rate
Voltage-gated potassium channel activity	KCNH1, HCN1, KCNS1, KCNAB3, KCNC3, KCNE2, KCNH7, CNGA4, KCNG4, KCNH5	10.94	2.75E-07	3.86E-04
Potassium channel	KCNH1, HCN1, KCNS1, KCNC3, KCNE2, KCNH7, KCNG4, KCNK12, KCNH5	8.29	1.24E-05	0.016
Anterior/Posterior pattern specification	ARC, HOXA2, HOXB2, HOXB8, HOXB5, VANGL2, HOXA10, HOXB9, ZBTB16, GLI2	8.04	3.96E-06	0.0066
